# Adherence to Azathioprine/6-Mercaptopurine in Children and Adolescents with Inflammatory Bowel Diseases: A Multimethod Study

**DOI:** 10.1155/2020/9562192

**Published:** 2020-02-25

**Authors:** Mervat M. Alsous, Ahmed F. Hawwa, Cameron Imrie, Andras Szabo, Eman Alefishat, Rana Abu Farha, Mohammad Rwalah, Rob Horne, James C. McElnay

**Affiliations:** ^1^Clinical and Practice Research Group, School of Pharmacy, Queen's University Belfast, Belfast, UK; ^2^Department of Clinical Pharmacy and Therapeutics, Faculty of Pharmacy, Applied Science Private University, Amman, Jordan; ^3^Department of Pharmacy Practice, Faculty of Pharmacy, Yarmouk University, Irbid, Jordan; ^4^University Hospital Southampton NHS Foundation Trust, Southampton, UK; ^5^Altnagelvin Area Hospital, Northern Ireland, UK; ^6^Royal Belfast Hospital for Sick Children, Northern Ireland, UK; ^7^Department of Biopharmaceutics and Clinical Pharmacy, Faculty of Pharmacy, The University of Jordan, Amman, Jordan; ^8^Department of Pharmacology, College of Medicine and Health Science, Khalifa University, Abu Dhabi, UAE; ^9^Paediatric Gastroenterology, Queen Rania Hospital for Children, Royal Medical Services, Amman, Jordan; ^10^Centre for Behavioural Medicine, UCL School of Pharmacy, University College London, London, UK

## Abstract

**Background:**

Measurement of the degree of adherence is a key element for the evaluation of treatment efficacy and safety; thus, adherence plays an important role in clinical research and practice. The aim of this study was to investigate medication adherence in children with inflammatory bowel disease (IBD) utilizing a multimethod assessment approach. A further aim was to examine factors that can influence adherence within this population.

**Methods:**

Medication adherence in 47 children (age range 3 to 17 years) with IBD in three centers in Northern Ireland and Jordan was assessed via subjective (parent and child versions of the Medication Adherence Report Scale (MARS) specific questionnaire) and objective methods, that is, high-performance liquid chromatography (HPLC) determination of the 6-mercaptopurine (6-MP) and azathioprine (AZA) metabolites in packed red blood cell samples taken during a clinic visit. Beliefs about prescribed medicines were also assessed in parents/guardians using the Beliefs about Medicines Questionnaire (BMQ).

**Results:**

An overall nonadherence to AZA/6-MP therapy in children with IBD was found to be 36.17% (17 out of 47 patients were classified as nonadherent using at least one of the assessment methods). A total of 41 patients (91.1%) were classified as adherent to AZA or 6-MP using the blood sampling, while adherence rates using the MARS questionnaire completed by children and parents/guardians were 60.6% and 72.7%, respectively. The latter provides a more longitudinal measure of adherence. Child self-reported nonadherence rates were significantly higher than parent/guardian reported rates (*p*=0.013). Binary logistic regression analysis identified age to be independently predictive of adherence, with adolescents (children aged ≥ 13 years old) more likely to be classified as nonadherent. Regarding the BMQ, when parental/guardian necessity beliefs outweighed concerns, that is, higher scores in the necessity-concern differential (NCD), adolescents were more likely to be classified as adherent.

**Conclusion:**

Results provide evidence for ongoing adherence challenges in the paediatric population with IBD. It is recommended that parents/guardians (particularly of older children) and older children themselves, should receive enhanced counselling and education about their prescribed medicines.

## 1. Introduction

Inflammatory bowel disease (IBD) describes a range of immune-mediated disorders resulting in chronic and intermittent gastrointestinal inflammation [1]. Symptoms of IBD include abdominal pain, recurrent diarrhea, weight loss, fever, lethargy, anorexia, and puberty delay in children [2]. Treatment of IBD includes a range of oral medications such as anti-inflammatories, immunomodulators, corticosteroids, and antibiotics [3]. Management of the disease may also involve the use of over-the-counter medications such as vitamins, iron and mineral supplements, dietary changes, infusions, or even surgery [3, 4]. Consequently, patients may have a complex medication regimen which can be overwhelming and give rise to concerns regarding adherence.

Adherence is defined as the degree to which patient medication taking behaviour corresponds with agreed healthcare providers' recommendations [5]. Adherence to treatment in IBD is a key element in the achievement of decreased disease activity, maintenance of periods of remission, and the achievement of adequate nutrition for the patient [5, 6]. Very few studies have examined adherence rates in children with IBD, and research in this area is largely restricted to the adult population [7]. Adherence in chronic paediatric diseases is a complicated matter as it can involve the whole family. In younger children, the parent or guardian is responsible for the administration of medications and ensuring that the disease is managed appropriately; however, as the child gets older, responsibility for taking medication transitions from parent to the older child. This latter transition has been shown to have a negative influence on adherence [8]. A systematic review reported a lack of medication adherence data in general in patients with IBD and indicated the need for further studies to investigate the impact of treatment adherence on clinical outcomes in these patients [9]. Despite the lack of a firm link between health outcomes and the level of adherence in children and adolescents with IBD, the adult literature suggests detrimental consequences as a result of nonadherence to the IBD regimen, with patients who are nonadherent being up to 5.5 times more likely to experience a relapse in symptoms [10]. Studies have suggested that many lifelong health management behaviours are established during adolescence and therefore understanding the factors that impact adolescents' adherence to their IBD regimen could potentially be beneficial in improving lifelong IBD management [11].

During the adolescent years adhering to IBD treatment has been reported to be particularly problematic [12]. Mackner and Crandall reported medication adherence rates of 38% and 48% in a paediatric IBD sample according to parent and adolescent reports, respectively [13], while others have reported adherence rates as low as 25–35% in adolescents [14, 15]. As management of medicines in children and adolescents involves both parents and the patients themselves, feedback on adherence is commonly collected from both parties as an indirect measure of adherence. Barriers to medication adherence in adolescents with IBD include drug side effects, negative medication beliefs [16], and cognitive and physical developmental factors [17]. Studies in adults with IBD [18] and other long-term conditions [19] have also shown that nonadherence is related to patient beliefs about the treatment, for example, how they judge their personal need for a particular medication(s) relative to their concerns about potential adverse effects.

Hommel et al. [20] showed that in children with IBD, the nonadherence rate for azathioprine (AZA)/6-mercaptopurine (6-MP) was 6% using subjective assessment and 46% using biological assays [20]. Since self-report is likely to overestimate adherence, it is therefore generally recommended that multimethod approaches are used to assess medication taking behaviour [21, 22].

AZA and 6-MP are immune-suppressants with short half-lives (3 and 1.5 hours, respectively) and therefore measuring their metabolites, that is, 6-thioguanine nucleotides (6-TGNs) and 6-methylmercaptopurine (6-mMP), is a more consistent direct method for adherence assessment and for therapeutic drug monitoring. Intracellular accumulation of AZA/6-MP metabolites occurs over a period of 2–3 weeks and therefore provides an indicator of long-term adherence [23]. While 6-TGN is considered the most active metabolite of the thiopurines [24], 6-mMP is considered responsible for side-effects of thiopurine therapy [25].

The primary aim of the present study was to assess medication adherence in children/adolescents with IBD utilizing a multimethod adherence assessment approach. A further aim was to investigate factors that may affect medication adherence within this patient group.

## 2. Methods

The study was approved by the research Committees in Royal Medical Services in Jordan (reference number: TF 3/1/EC/3719/) and the office of Research Ethics Committees Northern Ireland (ORECNI) (reference number: 11/NI/0100). Children with IBD, aged ≤17 years and who were receiving AZA/6-MP for at least one month were invited to participate in the study. Patients were excluded from the study if the parent/guardian or child refused participation in the study or if the child had developmental delay as judged by his/her physician. Recruitment of children was conducted in two medical centres in Northern Ireland (the Royal Belfast Hospital for Sick Children (RBHSC) and Altnagelvin Area Hospital (AAH)) and one centre in Jordan (Queen Rania Al Abdullah Hospital for Children-Royal Medical Services). In Northern Ireland, eligible parents and their children were invited to participate in the study by sending a letter ahead of the clinic visit while in Jordan they were approached at the same day of the clinic. Informed written parental consent to participate in the research was obtained by a research nurse for each child together with child assent in children ≥6 years old.

For each recruited patient, data on patient demographics, current medications and medical history were obtained from his/her medical files. An aliquot of whole venous blood (one sample per patient; 2 mL volume) was taken from a routine clinical blood sample withdrawn from the child during attendance at routine outpatient clinic. All blood samples obtained from patients were processed into plasma and packed RBC samples and stored at−80°C prior to analysis using a validated HPLC analytical method [26] modified as shown below.

### 2.1. Questionnaires Administered to Patients and Their Parents/Guardians

The following questionnaires were provided to patients and their parents/guardians recruited into the study for self-completion at the clinic, as follows:

#### 2.1.1. Medication Adherence Report Scale (MARS; Child and Parent/Guardian Versions) Questionnaire

The original parent and child versions of the Medication Adherence Report Scale (MARS) specific questionnaire were utilized to assess adherence of patients aged ≥ 11 years recruited in NI [27] while validated Arabic translated versions [28] were utilized in Jordan. Both versions have been shown to have good reliability [27, 28]. The parent version has 6 questions while the child version has 5 questions. In each version, item scores were summed and scaled to result in scores ranging from 1 to 5. Higher scores indicate higher levels of self-reported adherence. In the present study, a conservative 90% cut-off point for adherence was used; that is, a participant was considered to be adherent if the MARS score was ≥ 4.5 out of 5 [28, 29].

#### 2.1.2. Beliefs About Medicines Questionnaire (BMQ) Specific

Beliefs about prescribed medicines were assessed in parents using the Beliefs about Medicines Questionnaire (BMQ) [30]. BMQ scores can be categorized into two subscales, that is, necessities and concerns. Views expressed about the necessity of the medication for maintaining or improving health describe the ‘necessity beliefs' whereas beliefs about the potential adverse effects of taking medication are incorporated into the ‘concerns' subscale [30]. The original English version of the BMQ was utilized for patients recruited in NI, while the validated Arabic version was utilized for patients recruited in Jordan [28].

There are 11 statements in the questionnaire each being coded as either a necessity (*n* = 5) or a concern (*n* = 6) and participants indicate their degree of agreement with each individual statement on a 5-point Likert scale, ranging from strongly disagree to strongly agree. The total necessity and concern scores are calculated separately before being compared to see if the participant's overall view of their medications is that of necessity or concern. The scores of each necessity and concern scale were scaled to give scores ranging from 1 to 5. Higher scores indicate stronger beliefs in the perceptions represented by the scale. The balance of concerns relative to necessity is known as the necessity-concern differential (NCD). When the NCD is positive, necessity beliefs outweigh concerns and conversely when negative, concerns outweigh necessity beliefs [31].

#### 2.1.3. Measurement of AZA/6-MP Metabolite Concentrations in Packed Red Blood Cell Samples

A sensitive, selective, and high-performance liquid chromatography (HPLC) method that was developed and validated in our lab previously [26] was utilized for determination of 6-MP metabolites after being modified as follows. The sample preparation step involved addition of 150 *μ*l of water and 100 *μ*l dithiothreitol (75 mg/mL) to the 100 *μ*l of packed RBCs. The sample was vortexed for 1 minute after which 50 *μ*l of perchloric acid (700 mL/L) was added and vortex-mixed for a further 30 seconds. After centrifugation at 13,000 × *g* for 15 minutes, the clear supernatant layer was removed and heated for 45 min at 100°C using a heating block. An aliquot of 700 *μ*L of water was added and then vortex-mixed for 10 seconds before being transferred to (MCX) SPE cartridges (1 ml/30 g; Waters®). The final eluent was then dried for 20 minutes under a stream of nitrogen at 37°C and reconstituted in 100 *μ*L 0.05 M NaOH with vortex mixing for 1 minute. Samples were then subjected to HPLC with UV detection (322 nm and at 342 nm) using an Atlantis® (T3) dC18 column [150 mm × 4.6 mm; particle size, 3 *μ*m; Waters] protected with a guard cartridge of similar chemistry (20 mm × 4.6 mm; particle size, 3 *μ*m; Waters). The mobile phase solutions were degassed and filtered prior to use. Mobile phase A: 97% (0.02 M) phosphate buffer pH 2.25. Mobile phase B: acetonitrile. The mobile phase flow was a gradient (1 mL/minute) from 97% to 80% mobile phase A over 14 minutes followed by 11 minutes reequilibration with 97% mobile phase A. The assay limits of quantification for 6-mMPNs and 6-TGNs were 3.75 and 0.5 *μ*M, respectively. Concentration ranges covered by the assay validation (ICH guidelines) were 3.75–300 *μ*M for 6-mMPNs and 0.5–20 *μ*M for 6-TGNs.

Analysis of blood samples was conducted at Queen's University Belfast for patients recruited in NI and at the Pharmaceutical Research Center, Jordan University of Science and Technology for blood samples obtained from patients in Jordan.

#### 2.1.4. Triangulation of Adherence Assessment Data

Adherence to thiopurines was assessed using a combination of subjective (MARS) and objective (metabolite concentrations). Adherence classification of individual patients was as follows:

- If score was <4.5 using the parent/guardian MARS questionnaire, then the patient was deemed nonadherent.
- If score was <4.5 using the child MARS questionnaire, then the patient was deemed nonadherent.
- Levels of AZA/6-MP metabolites in packed RBCs were subjected to cluster analysis [29, 32]. The pattern of 6-mMP and 6-TGN metabolite concentrations in the IBD patients was investigated and patients were grouped according to their metabolite levels. The 20^th^ percentile was used as a cut-off point. Patients who had both 6-TG and 6-mMP concentrations below the threshold (20^th^ percentile) were classified as nonadherent [29].

Through triangulation of results from the different subjective and objective approaches, a patient was deemed as nonadherent if any of the assessment methods identified the patient as nonadherent.

### 2.2. Data Analysis

Descriptive statistics were used to describe the sample; the continuous variables were described using mean and standard deviation (SD); categorical variables were described using frequency and percentages. Group differences (adherent versus nonadherent) were explored using the Mann Whitney *U* test for continuous variables. Categorical variables were analyzed using Chi-squared analysis. If the expected frequency fell below 5, Fisher's exact test was employed. The Kappa (*κ*) coefficient was used to assess the magnitude of agreement between each of the methods of adherence assessment. Univariate analysis was used to explore the relationship between adherent and nonadherent patient groups and patient age, gender, disease duration, and beliefs about medicine. Binary logistic regression was then utilized to determine independent predictors of adherence to thiopurine therapy.

All analyses were carried out using SPSS® software version 22. The significance level was set at 0.05.

## 3. Results

### 3.1. Patient and Disease Characteristics

Forty-seven eligible patients (*N*. Ireland, *n* = 31; Jordan, *n* = 16) agreed to take part in this cross-sectional study. Response rates are of course a challenge with all clinical trials since only those patients/parents who provide appropriate consent can join the research. In the present research, eleven patients declined to take part in the research in Northern Ireland out of total 48 patients (31 patients from RBHSC and 17 patients from AAH). The main reasons for refusal by parents/guardians were no interest in research (8 patients), time limitation (1 patient), or travel plans already in place (1 patient), while one child refused to participate due to the anticipated pain associated with the finger prick for the DBS sampling. Six patients were not approached for various reasons, for example, transfer to another clinic (5 patients) or patient stopped taking the drug (1 patient). Response rate in NI was 64.6%.

On the other hand, 23 patients were eligible for recruitment into the study in Jordan: 16 patients agreed to participate while 7 patients refused to participate due to lack of interest in the research. The response rate in Jordan was 69.6%.

Thirty children (63.8%) were diagnosed with Crohn's disease, 14 (29.8%) were diagnosed with ulcerative colitis, and 3 patients (6.4%) had indeterminate colitis. The demographics and disease characteristics of the study sample are described in Table 1.

Thirty-six patients received AZA and 11 patients received 6-MP for the management of their IBD. The overall number of medications received by patients ranged between 1 and 7 medications. The recruitment fell about of target and therefore results should be considered indicative rather than definitive at this step.

### 3.2. Adherence Assessment

#### 3.2.1. Adherence Using Mars Specific (Parent and Child Versions)

**Figure 1:**
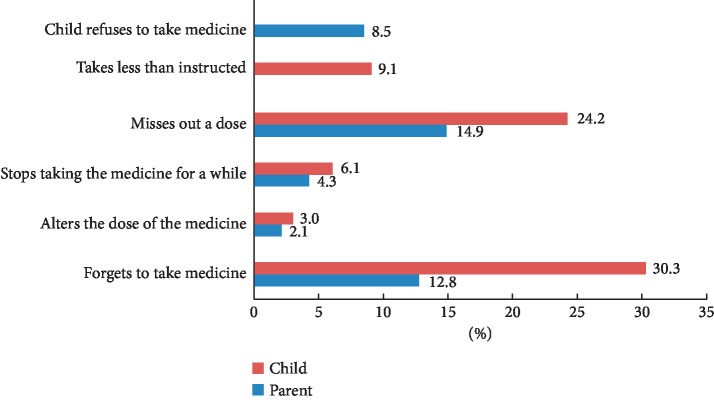
Percentage of patients who reported that they sometimes, often, or always engaged in nonadherent behaviours included in the MARS questionnaire.

Thirty-three patients (children ≥ 11 years old) completed the Mars child version while 47 parents completed the MARS parent version. Unintentional nonadherence was evident with 30.3% of patients reporting that they forgot to take their AZA/6-MP at least sometimes, often, or always. Intentional nonadherence was also prevalent with 24.2% of patients and 14.9% of parents reporting that they had decided to miss doses of AZA/6-MP. Fewer participants reported altering the dose of medication (one parent (2.1%); one patient (3%)). A few patients reported that they had stopped taking the medication (6.1%) or took less than instructed by their physician (9.1%) at least sometimes, often, or always (Figure 1).

The score distribution for the MARS questionnaire data is presented in Table 2. Using the parental MARS questionnaire scores, a total of 10 children (21.3%) were classified as nonadherent. On the other hand, across the 33 children who answered the MARS (child) questionnaire (those aged 11 years or above), 13 children (39.4%) were classified as being nonadherent.

#### 3.2.2. 6-MP Metabolite Concentrations in Packed RBC Samples

A total of 45 venous blood samples (processed into packed RBC samples) were obtained from the 47 patients recruited into the study; that is, two patients were not scheduled to provide a blood sample at the clinic in Northern Ireland.

**Figure 2:**
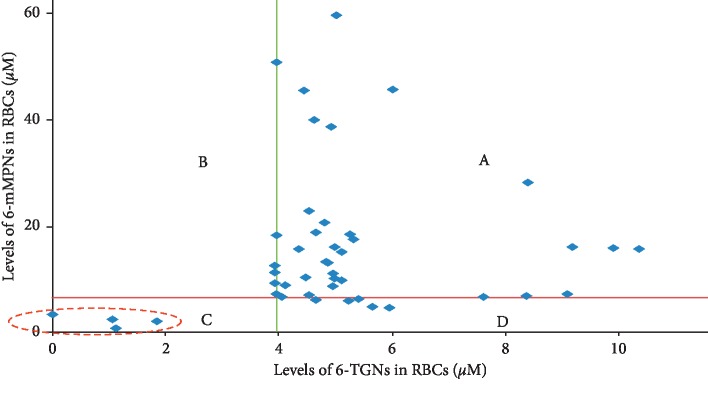
Scatter plot showing the four different clusters formed after clustering of the IBD study sample (*n* = 45) using 20th percentile of metabolite levels as cut-off point. Data for 6-mMPNs and 6-TGNs are the metabolite levels (adjusted per dose/SA). Cluster (A) (i.e., above 20% cut-off point) was characterized by high levels of 6-TGN and 6-mMP (adherent patients). Cluster (B) was characterized by high levels of 6-mMP but with low 6-TGN concentrations. Cluster (C) was characterized by low levels of both 6-mMP and 6-TGN (nonadherent patients). Cluster (D) patients have low levels of 6-mMP and high levels of 6-TGN.

Both 6-TGN and 6-mMP metabolite concentrations were measured for all patients who provided a blood sample. Based on cluster analysis and by using the 20th percentile of 6-mMP and 6-TGN metabolite concentrations as a cut-off point to segregate patients according to their adherence, 41 children (91.1%) were considered adherent and 4 children (8.9%) were classified as nonadherent (Figure 2).

#### 3.2.3. Comparison Between Different Methods of Adherence Assessment

In the present study, 30 patients had the same classification (adherent) and 3 patients were classified (nonadherent) irrespective of assessment measure used. The highest percentage of nonadherence, 39.4%, was observed using the child MARS questionnaire.

Finally, using all data available, a patient was classified as nonadherent if one of the measures was indicative of nonadherence. Using this triangulation approach, seventeen patients (36.2%) were deemed nonadherent in the present study.

Analysis of the interrater reliability agreement between the different adherence classification methods and the interrater reliability for the MARS (child) and MARS (parent) was found to be significant (Kappa = 0.463, *p*-value = 0.013) indicating moderate agreement between the two assessment methods. On the other hand, fair agreement was found between the packed RBC method and both the MARS parent and child questionnaires (Kappa = 0.256 and 0.229, respectively).

#### 3.2.4. Factors Affecting Overall Adherence to 6-MP

In the BMQ assessment, (10.6%) of the scores fell below the midpoint for the necessity scale (i.e., <2.5 out of a range of 1–5), indicative of low beliefs regarding the need for thiopurine therapy.

**Figure 3:**
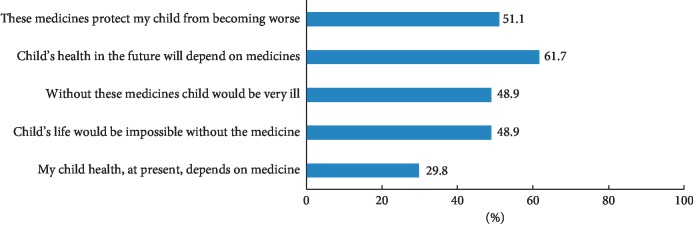
Percentage of parents indicating doubts about their child need for AZA/6-MP (responding strongly disagree/disagree/uncertain).

Approximately half of parents (48.9%) did not agree that without AZA/6-MP their child would be very ill. Sixty-one percent of parents did not support the statement that their child's health in the future will depend on their medicines. Almost one-third of parents (29.8%) expressed doubt or uncertainty that the health of their child at present depends on medicine (Figure 3).

**Figure 4:**
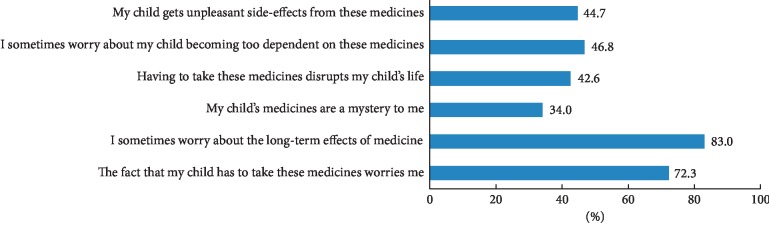
Percentage of parents responding (agree/strongly agree) to each concern item of the BMQ.

A large proportion of the parents (87.2%) scored above the scale midpoint for concerns regarding thiopurine therapy (i.e., > 2.5 out of 5).  Figure 4 provides a proﬁle of the concerns held about AZA/6-MP amongst the parents. The most prevalent were concerns about the long-term effects of the medicine (83%).

Univariate analysis was performed for all available continuous and categorical variables to compare data for children who were classified overall to be adherent with those who deemed as nonadherent to AZA/6-MP.

Of the factors studied in the univariate analysis, patient age was found to influence adherence (*p* < 0.01; i.e., adherence was lower in patients ≥13 years). In addition, there was also a significant difference in the NCD between adherent and nonadherent children who were older than 13 years (mean: 0.13 versus −0.26, respectively, *p*-value = 0.04). No significant associations were seen regarding patient gender, disease duration, type of disease, and total number of medications prescribed and overall adherence (*p*-value >0.05).

Logistic regression, using a backward stepwise (Wald) method, yielded a final model which included only one variable that was independently predictive of adherence, that is, the age of the patient, with age ≥13 years being associated with a higher chance of child being classified as nonadherent (Table 3).

## 4. Discussion

As is the case in any chronic illness, nonadherence to effective treatment in IBD carries a substantial risk to patient wellbeing [33, 34]. Due to the complexity of treatment of IBD [3, 4] patients often have to take multiple medications each day (>3 in the current cohort) which may be overwhelming for both child and parent. Better knowledge of factors which are linked to nonadherence to thiopurines in children will assist in the design of suitable practical interventions to promote adherence in this poorly studied population.

The use of a multimethod approach in assessing adherence helps to decrease bias associated with using a single method [35]. The current study utilized both subjective (self-reported questionnaires) and objective methods (measuring AZA/6-MP metabolites in packed RBCs) for adherence assessment. The nonadherence rate to oral medication in the paediatric IBD population in the USA has been found to be highly variable, with reported rates of 38%–66% using objective methods, and as low as 2% using self-reported adherence assessment [17, 20, 36–38]. In the present study, the percentage of patients classified as being nonadherent, taking into account both the subjective and objective assessments detailed, was 36.2%.

In the present study, cluster analysis of intraerythrocyte concentrations of 6-TGNs and 6-mMP was undertaken to identify patients who had no or low levels of both metabolites, which clearly indicates nonadherence to thiopurine medication [29, 32]. Upon the application of this analysis only 4 (8.9%) of the participating children were classified as nonadherent to AZA or 6-MP, that is, having their 6-mMPNs and 6-TGNs located within cluster C (Figure 2). No patient had measurable metabolite concentrations. This data indicates that all patients had been taking at least some doses of medication leading up to the clinic visit.

The self-reported questionnaires provide a more longitudinal assessment, that is, being not specific to the short period leading up to the clinic visit. The MARS is a validated tool to assess adherence in a different illness population [21, 22, 27–29] including IBD patients [18, 39] and can be used as a screening tool in clinical practice [39]. Child self-reported nonadherence (39.4%) was higher than parent/guardian reported nonadherence (21.3%), the difference being statistically significant (*p*=0.013). Children are perhaps more likely to answer the questions honestly due to their naive nature, while parents are more likely to present their child as adherent, not wishing to admit not following the doctor's instructions.

In the current study, there was no significant association between adherence classification and disease duration. This finding therefore is at odds with the hypothesis of longer disease duration leading to reduced adherence [3, 5]. A low mean disease duration of only 2.53 years and the low sample size may help explain this finding.

There are conflicting data on the role of a child's gender as a predictor of adherence to treatment [40]. In the present study, there was no association between gender and adherence classification, concurring with the findings in a number of other studies [5, 16].

Many studies have found that a high number of concomitant medications and multiple daily doses required in IBD management are reasons for nonadherence to oral treatments [16, 41–43]. In the present study, although the overall number of medications prescribed to each child ranged from 1 to 7, there was no significant association between adherence classification and the number of medications used.

The univariate and logistic regression analyses identified two factors which were associated with a child being classified as nonadherent, one relating to parents and one relating to children. The parental factor was the NCD score obtained via the BMQ. A previous study has shown that patients with high concerns and low perceived treatment necessity for IBD treatment were more likely to stop thiopurine treatment prematurely. Extra attention toward these patients might prevent premature discontinuation [44]. In the current study, NCD scores in parents of patients classified as adherent were positive in most cases; that is, necessity beliefs outweighed concerns. NCD scores were significantly higher (*p*=0.04) for parents of adolescent patients (≥13 years old) who were classified as adherent when compared with parents of patients in this age group who were classified as nonadherent. These data provide a clear insight into the potential utility of NCD assessment in parents, particularly those of adolescents, to facilitate identification of children at risk of poor adherence. Child age was the strongest predictor overall of adherence classification; it remained as the single statistically significant variable in the logistic regression model; that is, children ≥13 years old were more likely to be classified as nonadherent. This supports findings in previous studies which have reported that adherence decreases as age increases [14, 45] and is particularly problematic in adolescents [13]. Based on the present findings, it is therefore recommended that parents (particularly of older children who are responsible for their own medication) and older children themselves should receive enhanced counselling and education about their prescribed medications and the consequences of nonadherence.

## 5. Limitations

The sample size in the present study was small therefore reducing its statistical power; that is, the results should be considered as indicative rather than definitive at this stage. Future prospective trials, together with a meta-analysis of studies conducted to date, are recommended. Further work on psychosocial variables (e.g., socioeconomic status, parent/patient-physician interaction) is also required.

## 6. Conclusions

The overall rate of nonadherence to AZA/6-MP therapy in children with IBD in the present study was 36.2%. Only the child's age was found to be independently predictive of adherence.

The extent of nonadherence indicates that there is a wide scope to enhance adherence to AZA/6-MP in children with IBD; such interventions by healthcare providers should involve both parents and patients to increase adherence to more acceptable levels and address barriers to adherence, for example, doubts about personal need for AZA/6-MP and concerns about its side effects. Enhanced counselling/education about thiopurine therapy to reduce misunderstanding about the use of medication should be provided to adolescents and their parents since adolescents (≥13 years) are at higher risk of nonadherence to IBD medication.

## Figures and Tables

**Table 1:** Demographic and disease characteristics of the study sample (*n* = 47).

Parameters	IBD patients in Jordan (*N* = 16)	IBD patients in NI (*N* = 31)
Child age (years), median (range)	11 (3–15)	13.5 (3.9–17.4)
Child gender, *n* (%)		
Male	7 (43.8)	20 (64.5)
Female	9 (56.3)	11 (35.5)
Disease duration (years), median (range)		
Disease severity^#^, *n* (%)	2 (0.5–8)	2.5 (0.4–7.2)
Inactive	14 (87.5)	25 (80.6)
Mild/moderate	2 (12.5)	6 (19.4)
Severe	0 (0.0)	0 (0.0)
Number of medications, median (range)	4 (2–6)	3 (1–6)
Type of IBD, *n* (%)		
Crohn's disease	10 (62.5)	20 (64.5)
Ulcerative colitis	6 (37.5)	8 (25.8)
Indeterminate colitis	0 (0)	3 (9.7)
IBD medication type, *n* (%)		
Azathioprine	16 (100.0)	20 (64.5)
6-mercaptopurine	0 (0.0)	11 (35.5)
Thiopurine dose mg/kg, median (range)		
Azathioprine	2.0 (1.0–2.8)	2.1 (0.9–2.7)
6-mercaptopurine	—	0.9 (0.7–1.1)
Metabolite concentrations ^*∗*^		
6-TGNs (*μ*M), median (IQR)	4.55^*∗*^ (3.54–6.04)	4.85^†^ (4.31–7.08)
6-mMPNs (*μ*M), median (IQR)	10.18^*∗∗*^ (4.64–16.68)	13.23^††^ (8.09–18.34)

NI: Northern Ireland. IQR: interquartile range. ^#^The overall disease severity of each participating child was assessed by their physician, using the Paediatric Crohn's Disease Activity Index for those with Crohn's disease and using the Paediatric Ulcerative Colitis Activity Index for those with ulcerative colitis/indeterminate colitis. ^*∗*^6-TGN levels equivalent to 327.6 pmol/8 *∗* 10^8^ erythrocytes. ^*∗*^6-MPN levels equivalent to 746.5 pmol/8 *∗* 10^8^ erythrocytes. ^†^6-TGN levels equivalent to 349.2 pmol/8 *∗* 10^8^ erythrocytes. ^††^6-MPN levels equivalent to 970.2 pmol/8 *∗* 10^8^ erythrocytes.

**Table 2:** Distribution of the total scores for the MARS questionnaires completed by participating parents and children.

Measure	N	Total score mean (SD)	Total score range	Score indicating nonadherence	Number (%) of nonadherent patients
MARS (parent)	47	4.73 (0.32)	3.7–5	<4.5	10 (21.3)
MARS (child)	33	4.41 (0.59)	2.6–5	<4.5	13 (39.4)

**Table 3:** Predictor of adherence to AZA/6-MP using logistic regression.

Independent variable	B	SE	Odds ratio	95% CI	*p* value
Patient age	−0.38	0.15	1.47	1.10–1.96	0.009^*∗*^

B: regression coefficient; SE, standard error associated with the coefficient B. ^*∗*^*p* value < 0.05 (nonadherence coded 0, adherence coded 1).

## Data Availability

The data used to support the findings of this study are included within the article.
